# Prognostic impact of the loss of E-cadherin and *de novo* expression of N-cadherin at the invasive front of primary and recurrent oral squamous cell carcinoma

**DOI:** 10.3389/fonc.2023.1151879

**Published:** 2023-05-17

**Authors:** Samer George Hakim, Clara Taubitz, Steffen Hoppe, Daniel Steller, Dirk Rades, Julika Ribbat-Idel, Ubai Alsharif, Mohamed Falougy

**Affiliations:** ^1^ Department of Oral and Maxillofacial Surgery, Head and Neck Cancer Center, University Hospital Schleswig-Holstein, Lübeck, Germany; ^2^ Department of Oral and Maxillofacial Surgery, Helios Medical Center, Schwerin, Germany; ^3^ Department of Radiation Oncology, University Hospital Schleswig-Holstein, Lübeck, Germany; ^4^ Institute of Pathology, University Hospital Schleswig-Holstein, Lübeck, Germany; ^5^ Department of Oral and Maxillofacial Surgery, Dortmund General Hospital, Dortmund, and Faculty of Health, Witten/Herdecke University, Witten, Germany

**Keywords:** oral squamous cell carcinoma, E-cadherin, N-cadherin, epithelial-mesenchymal transition, survival, H-score

## Abstract

The epithelial-mesenchymal transition (EMT) is a biological mechanism in multiple pathophysiological diseases. Related alterations in cadherin expression play a crucial role in carcinogenesis, progression, angiogenesis, and immune response. EMT cells exhibit a transition from an epithelial to a mesenchymal phenotype (cadherin-switch). This process is characterized by the *de novo* development of N-cadherin (N-CAD), which replaces E-cadherin (E-CAD) and signifies an increased migratory capacity and malignant transformation. The cadherin switch is a hallmark of EMT and has been studied in various cancer entities. We predicted that the cadherin switch in the primary and recurrent oral squamous cell carcinoma (re-OSCC) tissues is an inherent characteristic of the tumor, affects the biologic behavior, and further reflects the post-recurrence survival outcome of these patients. Survival outcome was analyzed by calculating the post-recurrence survival of the high-risk group and correlating the standardized *h-score*-based IHC expression of both cadherin types with the clinical follow-up. 94 patients with re-OSCC were observed within the cohort. Tissue samples from both primary and recurring tumors were collected. There was a significant association between loss of E-CAD expression and both oral cancer-specific and overall survival, (HR=2.72, CI:1.50-4.95, *p*=0.001) and (HR=3.84, CI:1.93-7.63, *p*=0.001), respectively, for expression loss higher than 60%. There was no statistically significant correlation between N-CAD *de novo* expression and Overall, oral cancer-specific and disease-free post-recurrence survival. The current study clearly shows that cadherin-switch, identified as E-CAD loss and N-CAD *de novo* expression in the invasion front of a re-OSCC, appears to be an inherent histological hallmark that does not change from primary manifestation to recurrence within the same tumor, regardless of the form of adjuvant therapy used for the primary tumor. The loss of E-CAD expression in re-OSCC is an independent risk factor for poor survival, and may be used to stratify therapy and de/escalate the multimodal treatment.

## 1 Introduction

The epithelial-mesenchymal transition (EMT) is a cellular process involved in several pathophysiological conditions, such as inflammation, radiation damage, and wound healing. Related altered cadherin expression within this process plays a crucial role in tumorigenesis, tumor progression, angiogenesis, and tumor immune response (1, 2). EMT cells are characterized by a switch from an epithelial to mesenchymal phenotype (cadherin-switch). This change is associated with a distinct gene expression and post-translational regulation, leading to the repression of epithelial characteristics and the acquisition of mesenchymal features (3, 4).

During carcinogenesis and tumor invasion, epithelial cell-cell junctions, identified by intact E-cadherin staining (E-CAD), undergo rarefication, and partial, or total loss. These EMT cells acquire a fibroblast-like morphology and cytoarchitecture of mesenchymal cells. The *de-novo* expression of N-cadherin (N-CAD) is observed within this process, replacing E-CAD, and marks an increased migratory capacity and malignant transformation (5–8).

This cadherin-switch is widely considered a hallmark of EMT, and has been investigated in several cancer entities, predominantly in experimental mechanistic studies *in vitro* (9, 10). In the last two decades, it has been linked to cancer cell metastasis and invasion, and is suggested to be associated with an increased risk of recurrence, and poor survival, in various types of cancers, such as colorectal cancer, breast cancer, lung cancer, and oral cancer (11, 12). However, previous studies on E-CAD and N-CAD expression in oral cancer only focused on primary tumors and tried to correlate their findings with the clinical course of the disease.

Recurrent oral squamous cell carcinoma (re-OSCC) is a clinically aggressive form of OSCC. It carries the risk of uncontrolled local infiltration and the development of distant metastases. Patients with re-OSCC frequently undergo multimodal therapy, including salvage surgery and/or (re)radiation, chemo- or immune therapy. Despite the poor post-recurrence survival in general, some patients show a more favorable survival outcome. In contrast, others develop an early second recurrence after multimodal therapy and die shortly after that.

In order to understand the underlying mechanism of the biological behavior of re-OSCC, recent studies focused on the temporal changes in the mutation status of TP53, and revealed a correlation between TP53 within primary tumors and the post-recurrence prognosis (13, 14). Other studies investigated the role of altered tumor immune microenvironment (TIME) in re-OSCC as a sign of tumor cell evasion after primary radio(chemo)therapy (15, 16). As far as we know, none of them dealt with the E-CAD/N-CAD expression within the invasive front as a surrogate parameter for EMT, and its potential prognostic relevance in view of post-recurrence survival in patients with re-OSCC.

In an effort to transfer the EMT/cadherin-switch phenomenon as a histologic risk factor into a clinical context, we required a standardized evaluation and interpretation method for the EMT findings and the assignment of its degree to a specific risk profile and prognosis in a clinical setting.

We hypothesized that the cadherin switch in the primary and re-OSCC specimens is an inherent feature of the tumor, determines the biological behavior, and further indicates the post-recurrence survival outcome of these patients.

In order to investigate this hypothesis, we examined the immunohistological expression of both E-CAD and N-CAD in the primary and recurrent tumors of patients with OSCC in a prospectively maintained, single-center cohort. We evaluated the post-recurrence survival of this high-risk group and correlated the standardized *h*-score-based immunohistochemical expression of both cadherin types with the clinical outcome, identifying a threshold for post-recurrence survival.

## 2 Methods

### 2.1 Study population and data acquisition

**Figure 1:**
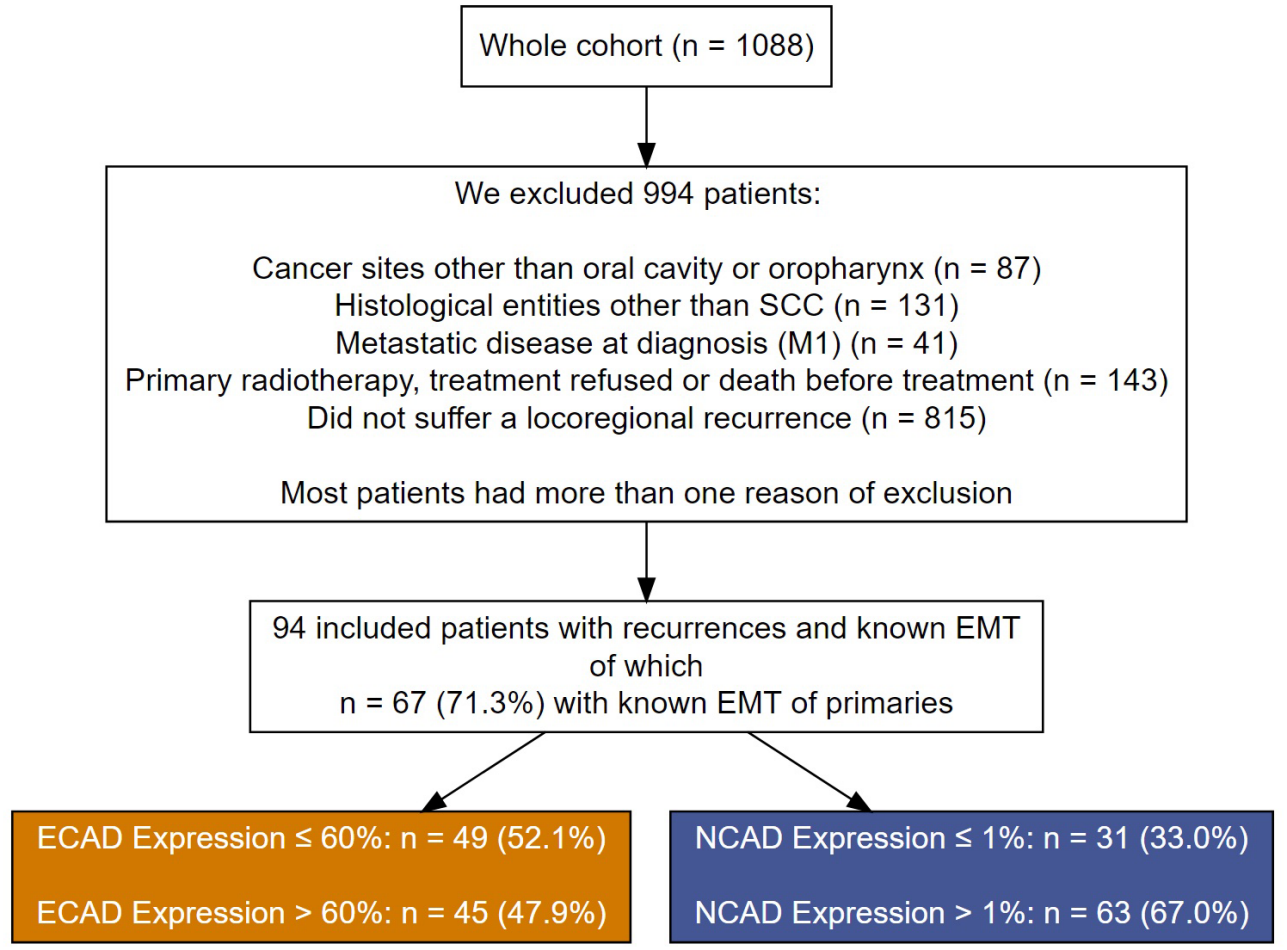
A flow chart showing patient exclusion criteria and their assignment to the different cutoff groups for E-CAD loss and N-CAD de novo expression in the recurrent OSCC.

From a cohort of 1088 cancer patients who presented at to the Department of Maxillofacial Surgery of the University Medical Centre of Lübeck, Germany, between 1992 and 2019, we selected 94 patients who suffered a recurrence of an OSCC. Those patients received with curative intent either surgery alone or combined with (chemo)radiotherapy (**Figure 1**). Patients were excluded if they had oropharynx carcinoma or histological entities other than SCC. Patients with metastatic disease at diagnosis, patients who refused treatment or died before therapy, and patients who did not have a locoregional recurrence were excluded.

All patients were enrolled in a strictly controlled recall system to ensure regular follow-up within the five-year post-therapeutic period (every three months in the first two years and every six months after that). Data were available at the beginning of the study and each follow-up. Demographic data, risk factors, clinical tumor characteristics, and treatment decisions were prospectively collected. The general condition of the patients, estimated using Charlson’s comorbidity score (17), the tumor stage, and other competing risk factors, were taken into account in the cohort analysis.

### 2.2 Tissue microarray

We obtained archived, formalin-fixed, paraffin-embedded tissue from surgically resected primary and recurrent oral cancer specimens from the Department of Pathology at the University Hospital of Lübeck. The specimens contained tumors and adjacent epithelial and mesenchymal tissues. The tissue samples were initially evaluated by conventional histology and categorized for tissue microarray (TMA). Specimens were available from 94 patients within the selected category, along with their clinical data, including TNM classification (according to the eighth edition of the UICC) (18), time of initial diagnosis and recurrence, adherence to follow-up, and censoring. All data was re-evaluated and double-checked by two experienced maxillofacial surgeons and a pathologist.

Tissue specimens were available from primary tumors, local recurrent tumors, and/or lymph node metastases (in cases with locoregional recurrence).

### 2.3 Immunohistochemical methods

Regions of interest (ROIs) were marked on hematoxylin and eosin-stained slides, and paraffin blocks were matched. Three 0.1 cm diameter (triplets) were punched from each tumor and organized in recipient blocks as TMAs. Sample cores that did not contain tumor tissue, had stain artifacts, or contained tissue folds were excluded (19).

Sections with 4*μ* thickness of formalin-fixed and paraffin-embedded tissues were deparaffinized, hydrated, heated in a steamer for 10 min with 10 mM sodium citrate (pH 6.0) for antigen retrieval, and washed in Tris buffer. Peroxide blocking was performed with 3% (v/v) H_2_O_2_ in methanol at ambient temperature for 15 min, followed by 10% bovine serum albumin in Tris-buffered saline-t at room temperature for 30 min. The slides were incubated with primary antibody at ambient temperature and washed with phosphate-buffered saline, followed by incubation with biotin-labeled secondary antibody for 30 min. Finally, the samples were incubated with a 1:40 solution of streptavidin–peroxidase for 30 min. Staining was developed with 0.05% (w/v) 3′,3-diaminobenzidine tetrahydrochloride, which had been freshly prepared in 0.05 mol/l Tris buffer at pH 7.6 containing 0.024% (v/v) H_2_O_2_ and then counterstained with hematoxylin, dehydrated, and mounted. Tissues from prostate cancer served as a positive control for E-CAD, and invasive lobular carcinoma (ILC) specimens were used as negative E-CAD control. Renal cell carcinoma and high-grade ovarian cancer were used as a positive control for N-CAD staining, and negative control slides were prepared by omitting the primary antibodies from the staining procedure.

TMA sections were stained using the automated Ventana BenchMark staining system and processed using the IViewDAB detection kit (both Roche^®^, Basel, Switzerland) (20).

Heat-mediated antigen retrieval was performed for 32 min at 92°C for both E-Cadherin and N-Cadherin staining using Cell Conditioning Solution 1 (CC1; #950-124, Ventana Medical Systems, Inc. Arizona, USA). As primary antibodies, we used a mouse monoclonal antibody from Roche^®^ (clone CDH1, dilution 0.314 μg/ml) for E-cadherin and a rabbit polyclonal antibody from Abcam^®^ (clone CDH2, dilution 1:100) for N-cadherin staining.

The Ventana iScan HT scanner was used to visualize the slides (Ventana, Tuscon, AZ, USA). The QuPath (University of Edinburgh, UK) image analysis software was used for the digital evaluation of the slides.

After running the TMA derrayer, all cores were identified, and the parameters were adjusted based on the staining features of each antibody (threshold, background intensity, and selection).

ROIs were annotated and categorized into different groups: tumor cells, immune cells, stroma, and others. The object classifier feature of the software was run to detect and analyze the numeric data of the different categories.

The *h-score*-based evaluation of immunostaining was applied to a maximum of 300. This was then generated by adding the percentage of strongly stained cells (weighted 3), the percentage of moderately stained cells (weighted 2), and the percentage of weakly stained cells (weighted 1), giving a possible range of 0 - 300 (21). The evaluation of the immunostaining was carried out, taking into account the percentage of positive staining tumor cells in relation to the entire examined tumor area.

E-CAD expression was classified as positive when membranous immunostaining was detected in epithelial tumor cells. To evaluate the loss of E-CAD staining within epithelial cells, an inverse estimation of the *h*-score-based evaluation was applied as 300 - E-CAD staining to normalize to N-CAD values and proportional vector development in both antibodies.

The N-CAD immunostaining was analyzed, considering the percentage of positive staining of tumor cells in relation to the entire examined tumor area. N-CAD expression was classified as positive when cytoplasmic or membranous immunostaining was detected in epithelial tumor cells.

The staining scores for both E-CAD and N-CAD were inserted as a continuous variable, and a cutoff was calculated for both categories based on the available sample size.

The optimal cutoffs for biomarkers were determined using the R package ‘Survminer’, which uses the maximally selected rank statistics in multivariate Cox’s proportional hazards regression models to provide the value of a cutoff that corresponds most strongly with the outcome. A cutoff value for E-CAD loss was established at 60% and for N-CAD expression at 1%, both based on the *h*-score.

### 2.4 Statistical analysis

Patients’ characteristics, as well as all analyses were stratified by E-CAD and N-CAD cutoffs. The level of E-CAD and N-CAD expressions were stratified by treatment of the primary tumor. All proportions were reported after excluding missing cases. All survival outcomes were measured from the time point of the diagnosis of the recurrence. The endpoint of overall post-recurrence survival (pr OS) was death from any cause. The endpoint of post-recurrence oral cancer-specific survival (pr OCSS) was death from oral cancer. The endpoint of post-recurrence disease-free survival (pr DFS) was the occurrence of local or locoregional recurrence. The patients were censored at the last follow-up. We estimated the median, 2‐ and 5‐year survival probabilities, and corresponding 95% confidence intervals (CIs) using Kaplan–Meier survival analysis for pr OS and cumulative events for pr OCSS and pr DFS. Cox’s proportional hazards regression models were used to estimate the adjusted hazard ratios (HRs) and corresponding 95% CI for E-CAD, N-CAD and associated risk factors. We introduced pr OCSS and pr DFS to the proportional hazards’ models in competing risks scenarios, which means that both outcomes had a death from any cause other than oral cancer as a competing risk. The assumption of proportional hazards was examined using Schoenfeld residual plots. All reported percentages were calculated after excluding missing values, and statistical analyses were performed using the R Statistical Software (version 4.0.4; R Foundation for Statistical Computing, Vienna, Austria). Results were considered significant at *p* ≤ 0.05.

### 2.5 Ethics

On admission, all participants signed consent forms allowing their data to be collected and used anonymously for academic research. The ethics review committee of the University of Lübeck approved the study (ID: 12-079A).

## 3 Results

### 3.1 Patient characteristics

Ninety-four patients with re-OSCC were identified within the cohort. Age at recurrence ranged from 53 to 72 years. Sixty-eight percent of local recurrences were associated with cervical lymph node metastasis, but only 8.8% were related to distant metastases. Eleven percent of the patients underwent radiochemotherapy primarily as an adjuvant treatment, and 50% only had radiotherapy. The remaining patients were treated only surgically.

Considering the missing cases, most patients had a positive smoking history (*n* = 69, 78%) and excessive alcohol consumption (*n* = 52, 60%). Nineteen patients (68%) demonstrated a safe resection margin (R0). In 9 (32%) cases, total resection was not possible (R1). Patients with distant metastases accounted for 8.8% of the total. The floor of the mouth (n= 39, 41%), the neck only (n= 15, 16%), the cheek/vestibule/retromolar (n= 13, 14%), and the anterior tongue (n=11, 12%) were the most frequently affected regions. Tumors were classified as rT1 in 24 (26%), followed by rT2 in 16 (18%), rT3 in 10 (11%), and rT4 in 28 (31%) patients. There were 63 (68%) patients with rN+ nodal status, compared to 29 (32%) patients who had no lymph node metastases (rN0). In the histopathological analysis, the majority of the patients (n=40, 59%) had moderately differentiated OSCC (G2). Proportional analysis was reported after excluding missing values (**Table 1**).

**Table 1:** A comprehensive descriptive analysis of all patients’ and tumor characteristics.

Variable	Overall	strata by E-CAD		strata by N-CAD	
	N = 94^1^	≤ 60% N=49 (52%)^1^	> 60% N=45 (48%)^1^	≤ 1% N=31 (33%)^1^	> 1% N=63 (67%)^1^
**Age at recurrence diagnosis**	63 (53-72)	63 (51-71)	63 (55-73)	62 (52-73)	64 (54-72)
Sex					
*Female*	30 (32%)	10 (20%)	20 (44%)	10 (32%)	20 (32%)
*Male*	64 (68%)	39 (80%)	25 (56%)	21 (68%)	43 (68%)
CCI score					
*0*	59 (63%)	32 (67%)	27 (60%)	21 (68%)	38 (61%)
*1 ≤*	34 (37%)	16 (33%)	18 (40%)	10 (32%)	24 (39%)
*Missing*	1	1	0	0	1
Smoking					
*Never*	20 (22%)	8 (18%)	12 (27%)	5 (17%)	15 (25%)
*Former or current*	69 (78%)	37 (82%)	32 (73%)	24 (83%)	45 (75%)
*Missing*	5	4	1	2	3
Alcohol consumption					
*None or moderate*	35 (40%)	12 (27%)	23 (53%)	10 (34%)	25 (43%)
*Excessive*	52 (60%)	32 (73%)	20 (47%)	19 (66%)	33 (57%)
*Missing*	7	5	2	2	5
Site of recurrence					
*Anterior tongue*	11 (12%)	4 (8.2%)	7 (16%)	5 (16%)	6 (9.5%)
*Cheek/vestibule/retromolar*	13 (14%)	6 (12%)	7 (16%)	3 (9.7%)	10 (16%)
*Floor of mouth*	39 (41%)	20 (41%)	19 (42%)	14 (45%)	25 (40%)
*Lip*	2 (2.1%)	0 (0%)	2 (4.4%)	0 (0%)	2 (3.2%)
*Neck only*	15 (16%)	9 (18%)	6 (13%)	7 (23%)	8 (13%)
*Oropharynx*	11 (12%)	8 (16%)	3 (6.7%)	2 (6.5%)	9 (14%)
*Palate*	3 (3.2%)	2 (4.1%)	1 (2.2%)	0 (0%)	3 (4.8%)
rT					
*rT1*	24 (26%)	12 (26%)	12 (27%)	5 (17%)	19 (31%)
*rT2*	16 (18%)	8 (17%)	8 (18%)	8 (27%)	8 (13%)
*rT3*	10 (11%)	5 (11%)	5 (11%)	3 (10%)	7 (11%)
*rT4*	28 (31%)	16 (34%)	12 (27%)	13 (43%)	15 (25%)
*rTx*	13 (14%)	6 (13%)	7 (16%)	1 (3.3%)	12 (20%)
*Missing*	3	2	1	1	2
rN					
*rN0*	29 (32%)	15 (31%)	14 (32%)	14 (45%)	15 (25%)
*rN+/x*	63 (68%)	33 (69%)	30 (68%)	17 (55%)	46 (75%)
*Missing*	2	1	1	0	2
rM					
*rM0/x*	83 (91%)	46 (98%)	37 (84%)	28 (90%)	55 (92%)
*rM1*	8 (8.8%)	1 (2.1%)	7 (16%)	3 (9.7%)	5 (8.3%)
*Missing*	3	2	1	0	3
Resection margins					
*R0*	19 (68%)	9 (75%)	10 (62%)	7 (54%)	12 (80%)
*R1/2/x*	9 (32%)	3 (25%)	6 (38%)	6 (46%)	3 (20%)
*Missing*	66	37	29	18	48
Grade					
*Well*	6 (8.8%)	2 (5.7%)	4 (12%)	2 (7.7%)	4 (9.5%)
*Moderate*	40 (59%)	18 (51%)	22 (67%)	16 (62%)	24 (57%)
*Poor*	22 (32%)	15 (43%)	7 (21%)	8 (31%)	14 (33%)
*Missing*	26	14	12	5	21

^1^Median (25%-75%); n (%).

All reported percentages were calculated after excluding missing values. Annotation, text and highlighting.

### 3.2 Characterization of immunohistological expression of E-CAD and N-CAD

**Figure 2:**
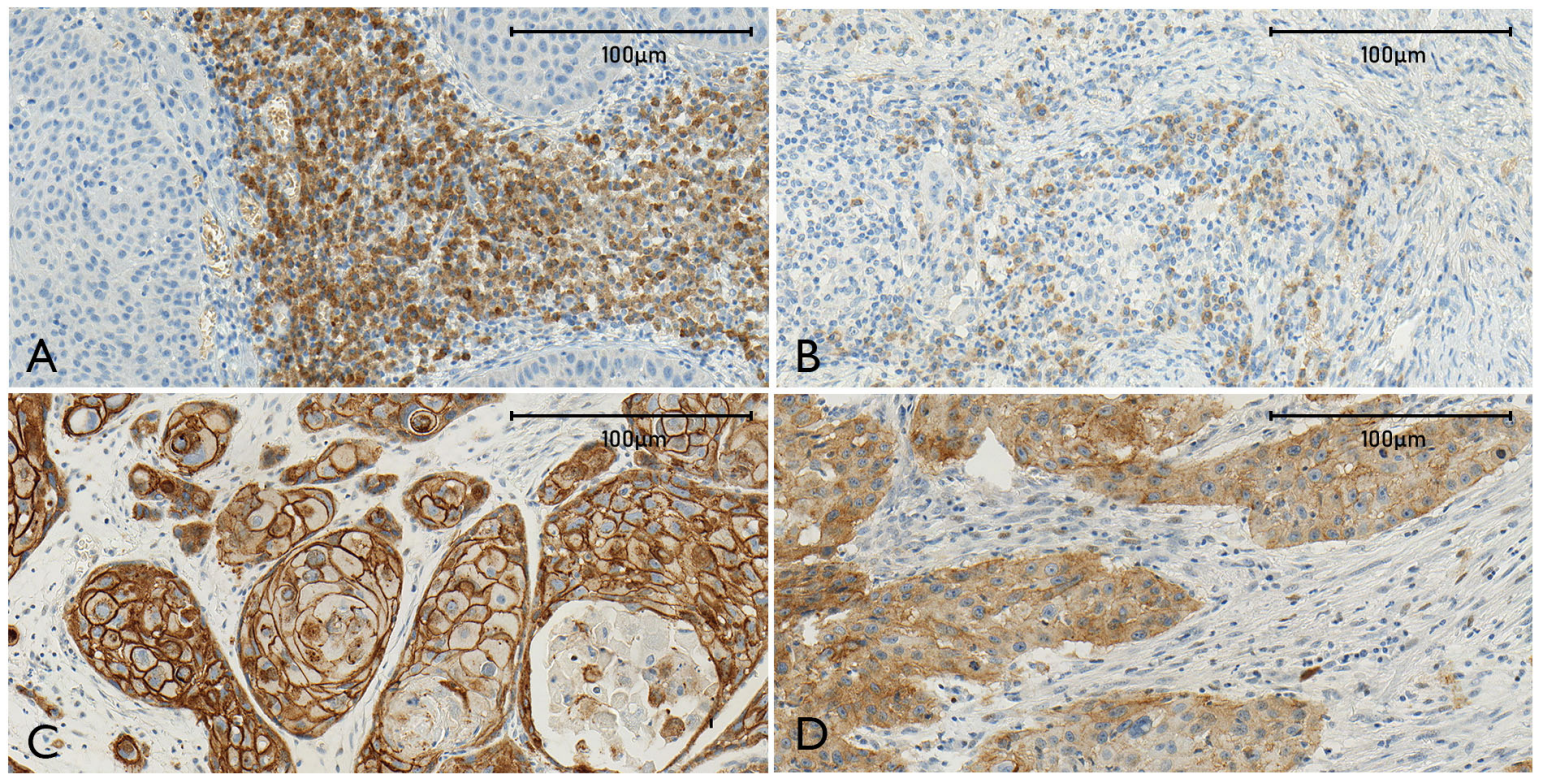
The combined figure **(A–D)** shows representative sections stained for N-CAD [**(A)** high expression, **(B)** low expression] and E-CAD [**(C)** widely maintained labeling of tumor cells, **(D)** E-CAD loss of membranous staining].

Normal epithelial areas adjacent to the malignant epithelium showed strong membranous E-CAD expression. A varying range of reduced intensity staining, and partial to total loss of membranous labelling was observed in both primary and re-OSCC (**Figure 2**). This phenomenon was evident in the suprabasal layer, epithelial cancer cells at the invasion front, and within the neoplastic epithelial nests in the stroma.

N-CAD was located in the cytoplasm and membrane in scattered tumor cells within the invasion front and in the stroma at variable intensities, and was independent of the grade of histological differentiation of the tumor. Adjacent areas with normal non-tumorous epithelial tissue did not demonstrate staining (**Figure 2**). The staining pattern for E-CAD and N-CAD was constant among the specimens, and consistent in primary and re-OSCC.

### 3.3 Expression of E-CAD and N-CAD in primary and recurrent tumors

E-CAD expression in primary tumors ranged from 43-62% (mean=53%), and was slightly higher in recurrence specimens (mean = 57%).

According to the *h-score*, the loss of E-CAD in epithelial tumor cells amounted to 4.95 (4.30-6.23) in primary tumors and increased in recurrent OSCC tissues to 5.91 (4.71-6.75) (Δ=6%)).

A similar effect was observed for N-CAD, since this showed a non-significant increase from the primary tumor tissues (mean=2.88% (0.29-4.09%)) to the recurrent tissues (mean=5% (1-7%)) with Δ = 2%).

Only a marginally increased difference in E-CAD loss in the non-irradiated subgroup was observed in the three subgroups in this analysis, surgery only, radiotherapy, and radiochemotherapy, and this was not significant. Otherwise, no difference in the E-CAD and N-CAD expression was identified among the subgroups.

The 5-year pr OS, pr OCSS, and pr DFS rates in the E-CAD group less than or equal to 60% were 30% (20-47%), 55% (42- 72%) and 41% (28-61%), respectively, while the rates in the group higher than 60% were 17% (8.9-33%), 79% (67-93%) and 61% (42-91%), respectively.

**Figure 3:**
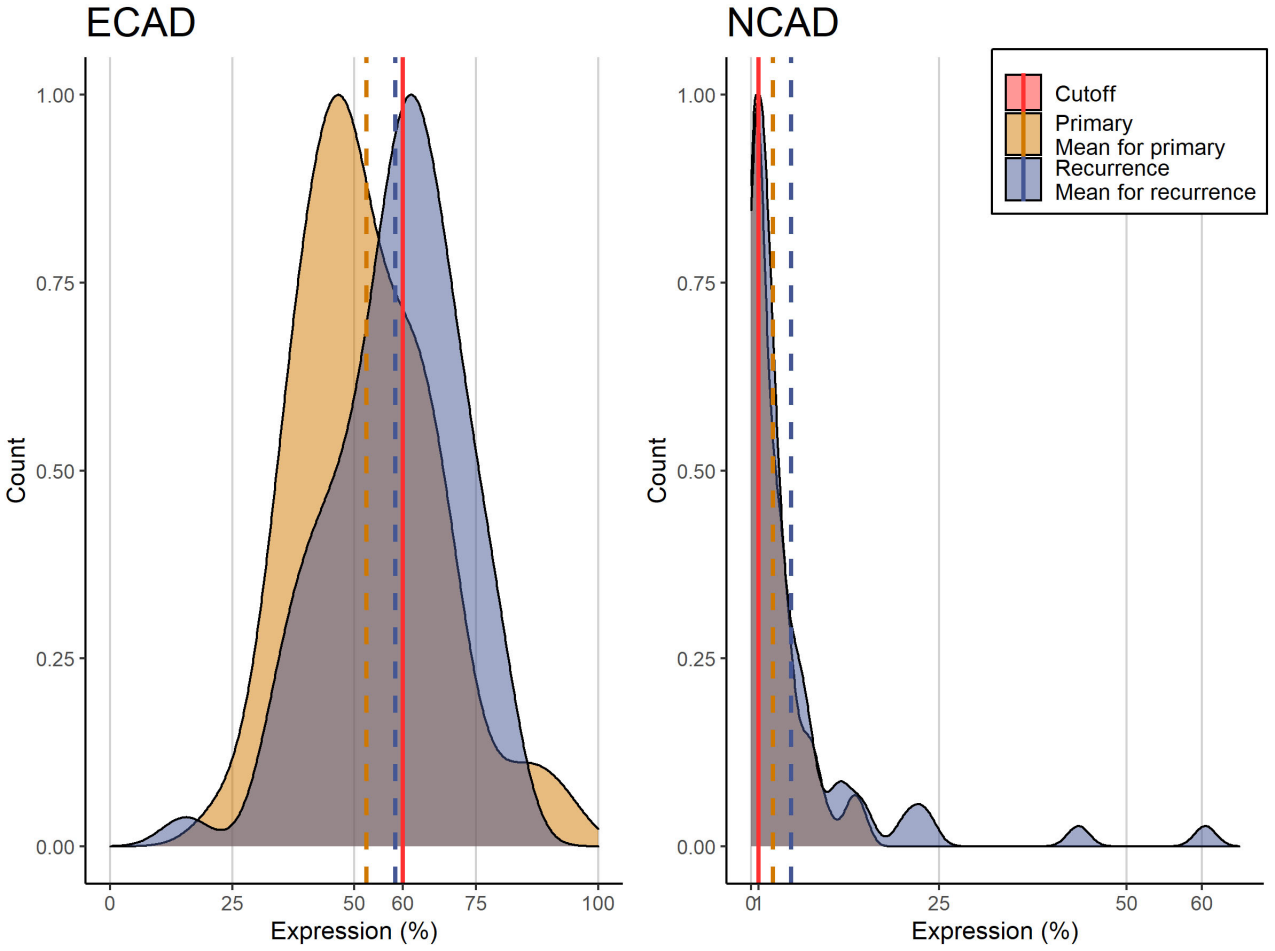
Histogram of E-CAD and N-CAD expression in primary and recurring tumors with the corresponding cutoffs.

**Figure 4:**
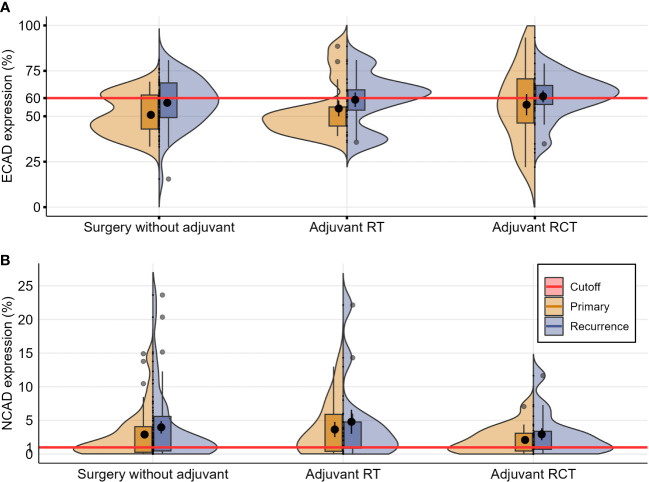
Violin plots depicting distributions of the numeric data for E-CAD **(A)** and N-CAD **(B)** expression in primary and recurring tumors, categorized according to the initial therapy after the primary diagnosis (each evaluated by the Wilcoxon test for paired samples, significant values with *p* ≤ 0.05).

In the N-CAD group less or equal to 1%, the 5-year pr OS, pr OCSS, and pr DFS rates were 19% (8.9-40%), 81% (67-96%), and 39% (22-70%), respectively, while the rates in the group more than 1% were 27% (18-40%), 58% (47-72%) and 51% (37-69%), respectively (**Tables 2**–**4** and **Figures 2**–**4**).

**Table 2:** E-CAD and N-CAD expression in primary and recurrence tumors divided by therapy of the primary tumor.

Characteristic	Overall, N = 94^1^	Adjuvant RCT N=21 (22%)^1^	Adjuvant RT N=15 (16%)^1^	Surgery without adjuvant N=58 (62%)^1^	p-value^2^
**ECAD Expression (Primary, %)**	53 (43-62)	56 (46-71)	54 (45-55)	51 (43-62)	0.6
*Missing*	27	8	2	17	
**ECAD Expression (Recurrence, %)**	57 (47-68)	57 (46-67)	57 (47-64)	57 (48-69)	>0.9
ECAD cutoff					>0.9
*≤ 60%*	49 (52%)	11 (52%)	8 (53%)	30 (52%)	
*60% <*	45 (48%)	10 (48%)	7 (47%)	28 (48%)	
**ECAD Expression (Δ, %)**	6 (-5-15)	5 (-9-17)	5 (-5-19)	7 (0-13)	0.9
*Missing*	27	8	2	17	
**NCAD Expression (Primary, %)**	2.88 (0.29-4.09)	2.10 (0.46-3.09)	3.65 (0.42-5.91)	2.91 (0.28-4.08)	0.8
*Missing*	27	7	2	18	
**NCAD Expression (Recurrence, %)**	5 (1-7)	4 (1-7)	4 (1-5)	6 (1-7)	>0.9
NCAD cutoff					>0.9
*≤ 1%*	31 (33%)	6 (29%)	5 (33%)	20 (34%)	
*1% <*	63 (67%)	15 (71%)	10 (67%)	38 (66%)	
**NCAD Expression (Δ, %)**	2 (-2-4)	1 (-2-3)	1 (-3-2)	3 (-1-5)	0.9
*Missing*	27	7	2	18	

RT, Radiotherapy; RCT, Radiochemotherapy. ^1^Mean (25%-75%); n (%).

^2^Kruskal-Wallis rank sum test; Pearson’s Chi-squared test; Fisher’s exact test.

**Table 3:** Survival events in the patients’ cohort according to the h-score-based cutoff by E-CAD and N-CAD expression.

Variable	Events by E-CAD		Events by N-CAD	
	≤ 60% N = 49 (52%)^1^	> 60% N = 45 (48%)^1^	≤ 1% N = 31 (33%)^1^	> 1% N = 63 (67%)^1^
Death from any cause				
*Alive or censored*	17 (35%)	9 (20%)	7 (23%)	19 (31%)
*Dead*	31 (65%)	36 (80%)	24 (77%)	43 (69%)
*missing*	1	0	0	1
Cause of death				
*Alive or censored*	17 (36%)	9 (21%)	7 (23%)	19 (32%)
*Death from oral cancer*	25 (53%)	32 (74%)	23 (77%)	34 (57%)
*Death from other causes*	5 (11%)	2 (4.7%)	0 (0%)	7 (12%)
*missing*	2	2	1	3
Post-recurrence disease-free survival				
*Censored*	31 (65%)	28 (67%)	22 (73%)	37 (62%)
*Locoregional recurrence*	17 (35%)	14 (33%)	8 (27%)	23 (38%)
*missing*	1	3	1	3

^1^n (%).

**Table 4:** Estimated survival durations by E-CAD and N-CAD cutoffs.

Variable	pr OS		pr OCSS		pr DFS	
	at 2 years	at 5 years	at 2 years	at 5 years	at 2 years	at 5 years
**Overall**	33% (25%-44%)	24% (17%-35%)	59% (49%-70%)	66% (57%-77%)	40% (30%-55%)	48% (36%-64%)
E-CAD loss cutoff						
*≤ 60%*	41% (29%-57%)	30% (20%-47%)	46% (34%-63%)	55% (42%-72%)	34% (22%-52%)	41% (28%-61%)
*> 60%*	24% (15%-41%)	17% (8.9%-33%)	72% (60%-87%)	79% (67%-93%)	52% (34%-78%)	61% (42%-91%)
N-CAD Cutoff						
*≤ 1%*	29% (17%-50%)	19% (8.9%-40%)	70% (55%-88%)	81% (67%-96%)	39% (22%-70%)	39% (22%-70%)
*> 1%*	35% (25%-49%)	27% (18%-40%)	53% (42%-67%)	58% (47%-72%)	40% (28%-58%)	51% (37%-69%)

pr OS, post-recurrence overall survival; pr OCSS, post-recurrence oral cancer-specific survival; pr DFS, post-recurrence disease-free survival.

These results indicate a constant grade of inherent initial cadherin switch within the primary tumors, which does not undergo alteration in the recurrence phase, and appears to be independent of the kind of adjuvant treatment applied (radio- or radiochemotherapy) between the initial diagnosis and recurrence.

The inverse correlation between E-CAD expression and the histological grade of the tumors in recurrent OSCC samples may be a notable observation. The majority of re-OSCCs (43%) that were classified G3 (poor-differentiated) showed a reduction of E-CAD expression of < 60%, while well-differentiated tumors mostly maintained a regular E-CAD expression (**Table 1**).

### 3.4 Association of E-CAD and N-CAD with clinical survival outcomes

We analyzed the survival events of the E-CAD and the N-CAD based on the *h-score* cutoff values shown in **Table 3**. Thirty-six patients (80%) who died showed a loss of E-CAD expression > 60%, and of this group of patients, 32 (74%) died from sequelae of tumor disease. The remaining patients died of other causes; in the group of patients with loss of E-CAD expression loss < 60%, 31 (65%) died, of whom 25 (53%) died as a result of tumor-related complications.

In the N-CAD group, 43 (69%) of the patients who died showed an expression of greater than 1%, and 24 (77%) of those who died showed an expression of N-CAD of less than 1%. Twenty-three (77%) patients in the group with lower N-CAD expression died due to tumor disease, and 34 (57%) of those with higher N-CAD expression.

There was a strong correlation between E-CAD expression loss in overall survival and oral cancer-specific survival. The HR increased by expression loss> 60 (high E-CAD loss) in comparison to low E-CAD loss in tumor cells (HR=2.72, CI:1.50-4.95, *p*=0.001) and (HR=3.84, CI:1.93-7.63, *p*<0.001), respectively.

**Figure 5:**
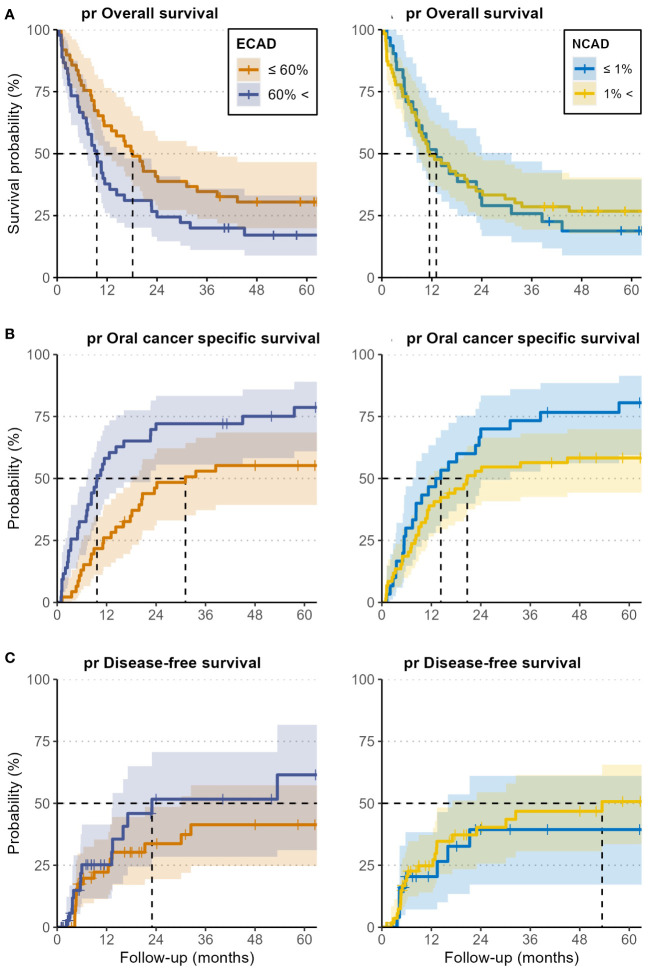
Kaplan-Meier curves for post-recurrence overall survival (pr OS) **(A)**, oral cancer-specific survival (pr OCSS) **(B)**, and post-recurrence disease-free survival (pr DFS) **(C)** of patients with high E-CAD loss and N-CAD de novo expression (blue curves), and for low E-CAD loss and negative N-CAD expression (yellow curves). Analysis was performed for both E-CAD and N-CAD separately. The cutoff was set at 60% for the loss of E-CAD and 1% for N-CAD de novo expression, both based on the h-score. All outcomes were calculated from the time of the first recurrence.

No statistically significant correlation was observed between the *de novo* expression of N-CAD and oral cancer-specific survival, oral survival, or post-recurrence disease free survival. Detailed results are given in **Table 5**, and the Kaplan-Meier curves are in **Figure 5**.

**Table 5:** Hazards ratios for different prognostic factors in recurrence specimens using the h-score-based cutoffs of E-CAD (60%) and N-CAD (1%).

Characteristic	pr OS			pr OCSS			pr DFS		
	HR^1^	95% CI^1^	*p*-value	HR^1^	95% CI^1^	*p*-value	HR^1^	95% CI^1^	*p*-value
**Age at recurrence diagnosis**	1.01	0.98-1.03	0.6						
Sex									
*Female*	—	—		—	—				
*Male*	0.97	0.47-1.96	>0.9	0.93	0.43-2.00	0.9			
Charlson comorbidity score									
*0*	—	—		—	—				
*1 ≤*	1.06	0.61-1.85	0.8	1.45	0.79-2.63	0.2			
Smoking									
*Never*	—	—		—	—				
*Former or current*	1.74	0.74-4.07	0.2	1.50	0.57-3.92	0.4			
Alcohol									
*None or moderate*	—	—		—	—				
*Excessive*	1.28	0.66-2.47	0.5	1.30	0.64-2.66	0.5			
rT									
*rT1*	—	—		—	—		—	—	
*rT2*	6.24	2.26-17.3	**<0.001**	6.00	1.96-18.4	**0.002**	4.11	1.45-11.6	**0.008**
*rT3*	5.85	1.98-17.3	**0.001**	8.29	2.53-27.1	**<0.001**	1.60	0.41-6.25	0.5
*rT4*	4.72	1.97-11.3	**<0.001**	5.15	1.85-14.3	**0.002**	1.43	0.51-4.00	0.5
*rTx*	4.22	1.55-11.5	**0.005**	7.48	2.41-23.3	**<0.001**	0.99	0.26-3.81	>0.9
rN									
*rN0*	—	—		—	—		—	—	
*rN+/x*	2.48	1.29-4.75	**0.006**	2.39	1.15-4.99	**0.020**	1.27	0.56-2.89	0.6
E-CAD expression (Cutoff)									
*≤ 60%*	—	—		—	—		—	—	
*> 60%*	2.72	1.50-4.95	**0.001**	3.84	1.93-7.63	**<0.001**	1.45	0.70-3.04	0.3
N-CAD expression (Cutoff)									
*≤ 1%*	—	—		—	—		—	—	
*> 1%*	1.23	0.68-2.21	0.5	0.90	0.47-1.73	0.8	1.60	0.69-3.73	0.3

^1^HR, Hazard Ratio; CI, Confidence Interval.

Significant values are given in bold.

Hazard ratios (HR) and 95% confidence intervals (CI) for overall, oral cancer-specific, and post-recurrence- disease-free survival were estimated using Cox proportional hazard regression analyses. Multivariate analysis for OS and OCSS revealed no statistically significant differences for possible risk factors such as sex, Charlson comorbidity score, smoking, or alcohol, with OS HR of 0.97, 1.06, 1.74, and 1.28, respectively, and OCSS HR of 0.93, 1.45, 1.50, and 1.30, respectively.

Post-recurrence overall and oral cancer-specific survival were all significantly lower in tumors larger than rT1. In terms of OS and PRFS, rT2 tumors had the poorest prognosis (OS HR= 6.24, CI 2.26-17.3, P= 0.001 and PRFS HR= 4.11, CI: 1.45-11.6, P= 0.008), and rT3 had the worst prognosis regarding OCSS (HR= 8.29, CI: 2.53-27.1, *p*= 0.001).

In patients with positive nodal status, both pr OS and pr OCSS were significant (pr OS HR= 2.48, CI: 1.29-4.75, *p*= 0.006 and pr OCSS HR= 2.39, CI: 1.15-4.99, *p*= 0.020).

## 4 Discussion

While diagnosis and treatment of primary OSCC are already established and anchored in specific related guidelines, management of re-OSCC is challenging interdisciplinary work. The clinical course of a recurrent OSCC differs substantially from that of a primary one in view of post-recurrence survival chances for many reasons. Although immune checkpoint inhibitors have been included in the latest treatment guidelines for recurrent oral cancer, response rates, and overall survival benefits stay low (22). Patients who underwent initial therapy for oral cancer suffered from various types of its sequela, including altered local anatomical structures and functional impairment in view of food intake, masticatory and speech deficiencies, and xerostomia (23). During initial chemotherapy, other general conditions, such as a reduction of renal and bone marrow function, aggravate these side effects and may further limit local and systemic treatment (24). It is, therefore, necessary to adjust the available treatment options to the general condition of these patients and stratify therapy according to their individual requirements and survival probabilities.

Among the various histopathologic prognostic markers that have recently been postulated to correlate with the survival outcome of patients with primary OSCC, we investigated the cadherin switch within the invasion front of re-OSCC. We evaluated the loss of epithelial E-CAD loss and the *de novo* expression of N-CAD in EMT cells using automated digital tools in a prospectively maintained cohort.

Numerous factors may regulate EMT and related changes in E-CAD and N-CAD expression in OSCC. These were mainly investigated in cell culture models and under standard conditions, which differ substantially from conditions in interacting multiple tissues/organs of patients. Such setting in particular doesn’t allow an estimation of the effect of E-CAD and N-CAD alteration on the post-recurrence survival outcome of patients with re-OSCC.

Recently, both radiation and platin-based chemotherapy have been supposed to affect the immunohistological features of OSCC cells [e.g., as an immunosuppressive TIME suggesting tumor immune evasion as one major factor, promoting tumor recurrence (15, 25)]. In the present study, however, the evaluation of E-CAD loss and N-CAD *de novo* expression showed unchanged values from the primary tumor specimens to their corresponding recurrences, regardless of the primary treatment (surgery only, surgery + radiotherapy, surgery + radiochemotherapy). This result confirms the cadherin homology in both primary and recurrent tumors in view of the cadherin switch after the initial therapy. These findings align with recent publications, ensuring coherence in the biological behavior and mutational synonymy in carcinoma cells at the invasion front (14).

Correlation clinical studies, functional experiments using cultured tumor cells, and transgenic mouse models have demonstrated that the function of E-CAD is replaced or overruled by the expression of mesenchymal cadherins, such as N-cadherin (26), and that the expression of E-CAD and N-CAD is mutually exclusive and reciprocal (27–29).

The inverse correlation of E-CAD expression with the tumor differentiation grade in our results is in accordance with early reports on the correlation of E-CAD expression with a grade of differentiation (G-status) in many tumor types, including squamous cell carcinoma of the head and neck in general (30), and especially in gingival carcinoma (31).

E-CAD is primarily involved in the deregulation of the extracellular matrix within the EMT process, and its loss at the invasion front is associated with poor DFS and OS (32–35). This aligns with our findings, since we assessed a certain degree of E-CAD loss throughout the tumor specimen series, albeit to a different extent.

E-CAD was gradually measured in the present study, and the values acquired were distributed across the range, and could therefore be integrated into a reliable regression model. Conversely, the *de novo* expression of N-CAD followed a fundamentally different pattern.

The N-CAD expression which marks a certain phase of EMT showed a scattered labeling pattern within the stroma. This was limited to a low expression level (< 5%) and was incoherent with the E-CAD loss range. For this reason, most previous studies only evaluated N-CAD expression in a descriptive manner and didn’t consider its change as a reliable marker for the grade of EMT. Our results align with these findings since the *de novo* N-CAD expression did not correlate with the three survival outcomes of the cohort patients. Similar findings were described by Hashimoto et al. They investigated the cadherin expression in an experimental mouse model and demonstrated the reduction of E-CAD-mediated cell-cell adhesion at the invasive front, but neither N-CAD nor the cadherin switch is a determinant of OSCC progression (36). The few available clinical studies addressing the correlation of E-CAD and N-CAD with the survival outcome in OSCC analyzed either data from primary OSCCs (37–39), the stage of primary disease as a variable (40), or compared tumor tissue with precancerous lesions of the oral mucosa as a control group (41). Two meta-analysis studies recently summarized the available literature on E-CAD expression and its correlation with the clinical data of patients with primary OSCCs (42, 43). Detailed features of some relevant previous studies (34, 37–39, 44–47) and a comparison to the present one are given in **Table 6**.

**Table 6:** Literature overview of comparable previous studies investigating E-cadherin and N-cadherin expression in OSCC, including study design, statistical evaluation methods, and adjustment to competing risks.

	Study design/ Type of cohort/samples size	Tumor manifestation	E-Cadherin Expression	N-Cadherin Expression	Statistical analysis	Outcomes
Present study	Single-center prospective cohort study (n=94)	Recurrent OSCC	Threshold-based evaluation (h-score)	Threshold-based evaluation (h-score)	Adjusted multivariate Cox proportional hazards’ regression models addressing competing risks	Post recurrence OS, OCSS, and DFS
Lòpez-Verdin et al. 2019	Case-Control study limited within 3 years of follow-up (n=40)	Primary OSCC	Low and high expression of E-Cadherin mRNA (not defined in detail)	Not investigated	t-test, Chi-square	E-Cad expression in OSCC compared to the control group
Hanemann et al. 2014	Comparative according to differentiation grading. Selected cases (n=71)	Primary OSCC	semi-quantitatively	Not investigated	t-test, Chi-square, log-rank	Kaplan-Meier-curves for OS and DFS
Fan et al. 2013	Retrospective cohort study (n=112)	Primary OSCC	Low and high expression of E-Cadherin classified semi-quantitatively	Not investigated	Cox multivariate regression adjusted for betel quid chewing, cigarette smoking, tumor size, TNM stage	Overall survival
Zhao et al. 2012	Retrospective cohort study (n=98)	Primary OSCC	semi-quantitatively	semi-quantitatively	Cox multivariate regression adjusted for TNM stage and N status.	Kaplan-Meier-curves for OS and DFS
Wang et al. 2009	Immunohistochemical Comparative Invasion front vs. center of the tumor	Primary OSCC	Low and high expression of E-Cadherin classified semi-quantitatively	Not investigated	Univariate	Overall survival related to E-Cadherin at the invasion front
Pyo et al. 2007	Retrospective Selected cases (n=71)	Primary OSCC	semi-quantitatively	Cut-off based (5%)	Cox multivariate regression adjusted for tumor differentiation, T status, N status, TNM stage, and mode of invasion.	Overall survival in primary OSCC with respect to P-, E-, and N-cadherins
Muñoz-Guerra et al. 2005	Retrospective Selected cases (n=50)	Primary OSCC	Low and high expression of E-Cadherin classified semi-quantitatively	Not investigated	Univariate and log-rank	Kaplan-Meier-curves for OS and DFS
Mattijssen et al. 1993	Retrospective IHC analysis in laryngeal and oral SCC. Selected cases (n=50)	Primary HNSCC	Low and high expression of E-Cadherin classified semi-quantitatively	Not investigated	Descriptive statistics Correlation analysis	Correlation with degree of differentiation and stage of disease

After a careful review of the related literature and to the best of our knowledge, the present study is the first to investigate recurrent OSCCs and correlate the post-recurrence survival outcomes with E-CAD and N-CAD expression. In the risk-adjusted hazard model, we demonstrated a significant decrease in the post-recurrence overall survival and post-recurrence oral cancer-specific survival once the *h-score* for E-CAD loss exceeded 60%. This IHC score thus represents an independent risk factor for poor post-recurrence survival in patients with re-OSCC.

The crucial difference between the results presented here and those of the related literature is primarily attributed to the evaluation method used for E-CAD and N-CAD expression. The main difference is that the cutoff used to define the threshold for a patient assignment was calculated differently. Previously, the cutoff was defined arbitrarily, primarily based on previous studies, and was subject to different interpretations by various, not always standardized immunohistological evaluation methods. The available clinical data from the prospectively maintained cohort also allows a bias-reduced and reliable risk-adjusted analysis since all relevant parameters were acquired at the baseline and can be reliably considered in the applied regression model.

Several experimental studies have shown that stimulation of OSCC cells *in vitro* with TGFβ1, a condition that can be clinically induced by radiotherapy (25), causes mesenchymal trans-differentiation accompanied by increased synthesis and deposition of both Lnγ2 and EMT along with a raised invasive capability (48, 49). In turn, EMT induces radioresistance, and increased cadherin-switch is supposed to limit the therapeutic efficacy of radiotherapy (50).

These findings and the results shown in the present study suggest that a therapeutic opportunity may be postulated by drug inhibition of EMT. This approach has already been investigated using metformin, which revealed the ability to inhibit EMT in OSCC *via* the mTOR/HIF-1α/PKM2/STAT3 pathway (51). Prospective randomized clinical trials are now warranted to evaluate the benefit of this therapy in patients with re-OSCC.

In summary, although the EMT-associated E-CAD expression has already been investigated in previous studies, 4 crucial differences are evident in the current one.

1. The current study deals with recurrent OSCC; previous ones investigated mostly primary tumors. The clinical course of a recurrent OSCC differs substantially from that of a primary one in view of post-recurrence survival outcomes.
2. We used a well-defined h-score-based threshold for E-CAD expression, which can be applied clinically in a standardized and reproducible way.
3. The initial E-CAD and N-CAD expression in primary OSCCs was compared to their recurrent tumors. This was performed in separate groups categorized according to the initial therapy (surgery alone, surgery + radio(chemo)therapy, definitive radio(chemo)therapy).
4. The clinical data in the present study are derived from a single-center prospectively maintained consecutive cohort, with initial patients’ data available at the baseline. This high-quality data set reduces potential data collection and processing bias as defined in the REMARK requirements criteria (52). The current epidemiologic analysis considered further all potential risk factors as competing risks. Thus, three endpoints, post-recurrence overall survival, post-recurrence oral cancer-specific survival, and post-recurrence disease-free survival, could be evaluated reliably.

One of the drawbacks of this study was the sample size. Another issue is that the EMT process is far beyond the cadherin-switch. The process is affected at multiple levels, such as cell signaling, epigenetic modification, post-translational modifications, and transcriptional control (53). The distribution of patients who received various treatments (surgery only, surgery + radiotherapy, surgery + radiochemotherapy) was also not equal.

## 5 Conclusions

The present study suggests that cadherin-switch in the sense of E-CAD loss and N-CAD *de novo* expression in the invasion front of re-OSCC seems to be an inherent histological hallmark that doesn’t change within the same tumor from primary manifestation to recurrence, regardless of the kind of adjuvant treatment for the primary tumor.

Using the automated evaluation of the *h*-score for IHC staining, we assessed strong evidence of a proportional correlation between E-CAD loss, post-recurrence oral cancer-specific survival, and post-recurrence survival in re-OSCC. The hazard ratio for post-recurrence survival outcome increased significantly by E-CAD loss of ≥ 60%. The *de novo* expression of N-CAD was limited and did not correlate with the post-recurrence survival outcomes.

The loss of E-CAD is thus an independent risk factor for poor survival in patients with re-OSCC and may be used to stratify therapy and de/escalate multimodal treatment. Targeting EMT, for example by inhibition, may therefore be a promising adjuvant treatment in patients with re-OSCC.

## Data availability statement

The datasets presented in this article are not readily available because the regulations of our local ethic committee do not allow data sharing . Requests to access the datasets should be directed to samer.hakim@uni-luebeck.de.

## Ethics statement

The studies involving human participants were reviewed and approved by the ethics review committee of the University of Lübeck approved the study (ID: 12-079A). The patients/participants provided their written informed consent to participate in this study.

## Author contributions

SH, MF: conceptualization. SH, JR-I, SH, CT: methodology. UA: formal analysis. SH, UA: investigation and visualization. SH, MF: writing/original draft preparation. SF, MF, UA: writing—review and editing. SH, DR: supervision and project administration. CT, SH, DS: Data curation. MF and UA contributed equally to the manuscript. All authors contributed to the article and approved the submitted version.
